# Porcelain Filling

**Published:** 1890-03

**Authors:** 


ARTICLE V.

### PORCELAIN FILLING.

Dr. Stoddard's method is thus described in the *Archives of Dentistry:*

As briefly as possible, I will give the method which we employ. There is nothing particularly novel about it to one used to porcelain work, but it seems to me that it is the most practicable in all cases of any that I have yet seen. Shape the cavity to general form desired, without making any undercuts. Leave the more careful trimming until the time for fitting the filling into place. (The specimen I pass around will show a tooth prepared in this manner, also the porcelain filling as it comes from the furnace, without any grinding). Take an impression of the cavity with Ash's modeling compound, using it in a stick the form of a pencil. Warm one end over a small flame, and thrust it into the cavity, taking care to get an accurate impression of the parts of the tooth immediately surrounding the cavity, as well as the cavity itself.

The color is now selected from the sample colors. Each sample corresponding in number to a body of that number. In this manner there is a certainty that the body, when baked, will be of the color desired.

Cast the impression without oiling it, and use the plaster paris very thin. In this manner an accurate model of the tooth and cavity may be obtained.

After separating, trim the margin of the cavity, in the plaster model, till it is slightly larger than the cavity in the natural tooth, to allow for shrinkage in baking, and make a slight undercut. Then mix the body of the desired number to the consistency of cream, pack into the cavity in the plaster model until full, and cover with a thin coating of enamel.

Place in the gas furnace, bake about two minutes. This biscuits the filling so that it may be removed from the plaster, otherwise, the model would melt when subjected to the intense heat in the furnace. Continue the baking for about six minutes longer, and the filling is fused.

When cool, it may be ground into place, set and polished in a surprisingly short time.

The manner of setting being virtually the same in all methods.

